# “CATHOLICOVID-19” or QUO VADIS CATHOLICA ECCLESIA: the Pandemic Seen in the Catholic Institutional Field

**DOI:** 10.1007/s41603-020-00114-2

**Published:** 2020-09-18

**Authors:** Emerson Sena da Silveira

**Affiliations:** 1 Post-graduation Program on Science of Religion (Religious Studies), Federal University of Juiz de Fora [Universidade Federal de Juiz de Fora - UFJF], Juiz de Fora, Minas Gerais (MG) Brazil; 2 https://www.ufjf.br/ppcir/

**Keywords:** Institutional Catholicism, Pandemic, CNBB, Heralds of the Gospel, CCR, public Catholicism, Catolicismo institucional, pandemia, CNBB, Arautos do Evangelho, RCC, catolicismo público

## Abstract

The aim of this paper is to understand, in a panoramic way, the ideas that some organized groups of Catholicism have expressed about the pandemic of the new coronavirus. We shall take as material for analysis, the web official pages of the following segments: Conferência Nacional dos Bispos do Brasil (CNBB) [National Conference of Bishops of Brazil], Heralds of the Gospel and Catholic Charismatic Renewal (CCR), especially from the moment when the first case of the disease caused by the Sars-Cov-2 virus and the health-social-economic emergency were checked. Catholic beliefs about Covid-19 and related themes (restrictive measures, social inequalities) show an intense and internal conflict of values or worldviews and lead to inquiring about the incidences of Catholicism in the public sphere. The qualitative-exploratory hypothesis demonstrates that the advancement of the new coronavirus has accentuated tension lines existing in the Catholic Church and indicates that there is an ongoing dispute between the various official segments about the correct intonation of the Catholic voice in Brazilian society. To raise responses to the proposed problem, the paper is based on a qualitative method, namely, partial review of the bibliographic productions of the religious studies and analytical mapping of the main official positions (editorials, speeches, notes, texts) proposed by the three Catholic segments aforementioned.

## Introduction

At the end of 2019, on December 31, a new virus of the corona family, SARS-CoV-2 (Severe Acute Respiratory Syndrome Coronavirus 2),^1^ was discovered in China, which causes a disease called Coronavirus Disease 2019 (COVID-19) by the World Health Organization (WHO). The patients infected with the new coronavirus may be asymptomatic, but a good percentage of cases (20 to 30%) may require hospital care for breathing difficulties, a small percentage of those affected (4 to 8%) may need treatment for respiratory failure (ventilatory support in an intensive care unit-ICU)^2^ and a smaller percentage (from 0.5 to 1.5%) may die (Bandeira and Carranza 2020).

In countries such as Brazil, which has been experiencing a sharp deterioration of republican institutions due to the neoliberal and anarcho-capitalist^3^ extreme right political action that came to power following the 2018 presidential elections, pandemic mortality reaches more poor, black, pardo Brazilian, and indigenous people, as well as social classes unassisted by state-public policies. Since the beginning of the serious health-social-economic crisis caused by the coronavirus, on February 25, 2020, President Bolsonaro has positioned himself in public, minimizing, despising, or debauching both the pandemic and the measures recommended by WHO and those that were taken by Brazilian governors/mayors. The mainstream media recorded President Bolsonaro’s embarrassing phrases when Brazil exceeded 5000 deaths on April 28. When asked about the seriousness of the pandemic, he replied to reporters at the door of the Palácio do Planalto: “So what? I am sorry. What do you want me to do? I am Messias but I do not perform miracles.”^4^

COVID-19 has spread quickly throughout the country, which has continental dimensions, reaching the 27 administrative units (26 states and one Federal District, headquarters of the national capital, and the five political-administrative regions, North, Northeast, Center-West, South, and Southeast), where 217 million inhabitants are distributed, six metropolitan regions with more than five million inhabitants, one of the largest Latin American metropolises (Sao Paulo, a 12 million people city). The very high urban poverty, the terrible environmental, economic, rural, and social indicators (HDI – the Human Development Index), however, caused the pandemic^5^ to affect the indigenous and black population (1% and 43% of the national population respectively),^6^ unemployed people, as well as those with very precarious occupation and without labor or social protection (13% or 29% of the economically active population respectively, more or less 13 and 31 million).

From March 2020 on, Brazil has adopted several measures of isolation and social detachment to contain this pandemic, as indicated by the WHO. Restrictive measures have been adopted by public managers, especially governors and mayors, implying the suspension of most economic, tourist, recreational, educational, and religious activities, among others, as well as prohibitions and/or restrictions on meeting and circulating in public spaces.

However, President Bolsonaro has been against all these measures, asking for the end of the quarantine, besides not adopting the recommended measures, hindering the transfer of public funds to fight the pandemic of COVID-19, etc.,^7^ barely wearing the mask and has vetoed, by decree, its use in prisons, commerce, and churches, participating in agglomerations in public spaces, hugging and handshaking other people, especially upon receiving his “flock” at the doors of the Palácio do Planalto. Many of his supporters come to him with religious banners, prayers, coming from charismatic Catholics, conservative Catholics, and Pentecostal and Neopentecostal Evangelicals (Werneck and Carvalho 2020). Many other situations have rekindled political polarizations, especially when the Brazilian president acts by suspending isolation measures and recommends the use of non-recommended medication for the treatment of COVID-19 (i.e., chloroquine) (Werneck and Carvalho 2020).

In this scenario of some clashes between the president’s, the governors’, and the mayors’ guidelines and those of WHO, some religious leaders, many of them Catholic, although not official ones, such as the CNBB (National Conference of Bishops of Brazil), aligned themselves with the President Jair Bolsonaro, especially regarding the interruption of services and the closing of the temples as a form of collaboration with the isolation measures (Bandeira and Carranza 2020).

On several occasions, pastoral elites and Evangelical and Catholic parliamentary elites have publicly defended the end or relaxation of restrictive measures, especially those that limited attendance to religious worship and services.^8^ In some pastoral lectures, many religious leaders minimized the pandemic and created a scenario of optimism in relation to the Federal Government, especially from the idea that Brazil would become more Christian and that President Jair Bolsonaro would be chosen by God for presiding over the nation (Bandeira and Carranza 2020).

The arguments used to contest restrictive measures are: the principle of religious freedom/freedom of expression, which is put forward as if it were absolute even in the face of a serious public health emergency, the dissemination of cure ads or promises of immunization against the virus, the idea that churches would be an “essential service” that would have a double task, i.e., spiritual and assistance, and the minimization of the effects of more stringent measures adopted by governors and mayors (Bandeira and Carranza 2020). The arguments in favor of restrictive measures used by religious groups include the valorization of science, public health policies guided by reflective-rational-political knowledge, the search for a balance between principles, without turning into absolute the freedom to come and go or to express/believe something to the detriment of health and education for all, and following pastoral guidelines from leaders more in tune with political and economic discussions that seek to understand and mitigate the multiple social, health, and economic impacts (poverty and inequality of wealth, income and race) accentuated by the advancement of COVID-19.

Amid the new coronavirus pandemic and the pandemonium of widespread social-economic conflicts, the official bodies of Catholicism—which is around 55% of the Brazilian population^9^—have reacted differently, or have concealed/belittled or even expressed social-religious concern. Searching the website portals of three official Catholic segments, CNBB, Heralds of the Gospel, and CCR-Brazil (Catholic Charismatic Renewal of Brazil), we shall focus on two types of documents in virtual media that mention COVID-19: official positions (notes/letters/editorials/pronouncements) or their absence, and news, produced by these groups or reproduced by others. The choice of these three groupings is due to the strength of their symbolic representativeness and their national organizational capacity: thousands of Catholic followers, lay people and priests, great communication structures, official recognition of the Holy See (Rome), and the claim to be the voice of Catholicism in Brazilian society. For reasons of space limitations and approach—centered more on conservative and reactionary movements, the important Catholic aspect of Liberation Theology, whose greatest ecclesial expression is the Basic Ecclesial Communities (CEBs), which has had so much importance in Brazilian history, has not been analyzed, especially between the late 1960s and the late 1990s (Rowland 2007; Dawson 2007). It can be said that, in the pandemic of the new coronavirus, this aspect of Catholicism, by developing a critical and theological-social stance linked to the search for justice and social equality, has aligned itself with the social and religious groups that adopt and defend scientific-sanitary protection measures and socioeconomic measures to protect the most vulnerable ones (black people, indigenous people, homeless population, unemployed people, precarious workers, etc.).

The beginning of the analytical mapping is on 26 February, the date of the official recognition of the first case, up to 5 July of this year.^10^ Keywords or semantic descriptors have been used in the internal electronic search engines for each site, including published documents.

The initial results show that the segments marked by academic literature as reactionaries and conservatives (Heralds of the Gospel and CCR-Brazil, respectively), tend to hide, minimize the impacts of COVID-19 and, when they do not, resort to strictu sensu religious procedures (prays, litanies, and prayers).

However, the official Catholic hierarchical organism, the CNBB, talks frequently to the public, expresses agreement with the papal concerns about the environment, social justice, care for the poor and indigenous people—close to those of the Catholicism of justice and social equality—recommends the adoption of measures by international health agencies and, finally, accepts restrictive measures to combat the new coronavirus. The scenario of conflicts between health guidelines and public policies around combating the pandemic has accentuated the disputes for an ideal representation of what “Catholicism” is in the public sphere between the instances of Catholic grouping, CNBB, the Heralds of the Gospels, and CCR-Brazil.

Two types of pandemic beliefs and ideas have been identified, personified in more conservative or more reactionary groups that are indifferent or underestimate the new coronavirus, approaching far-right/neoliberal governments like Bolsonaro’s, and in groups more attentive to the world health standards, the vast social-human impacts of the pandemic, thus keeping a distance from governments that despise the deadly COVID-19. In our argument, the emergence of the Covid-19 pandemic has enabled the formation of a field of forces into which conservative religious groups converge, who wish to limit some impacts of modern structures of state, culture, and society on moral and family values and reactionary religious groups as well, which seek to deny and combat aspects of the modern state, social and cultural structure, especially those related to sexuality and the family. Next, an overview of the Catholic Church shall be made, in order to describe the results of the analytical mapping of official positions (notes, speeches, texts).

## Catholic Church in Brazil: Trends and Conflicts

The presence of Catholicism in the Brazilian political-public sphere has been established since the processes of Republic foundation (1889) and the First Republican Constitution (1891–92) and follows, more or less, the socio-political-economic problems that emerge from the national and international contexts and Brazilian economic, political, cultural, and social changes (Dias 1996; Romano 1979; Souza 2002). It will not be possible within the scope of this paper to approach more deeply the relationship between the Catholic Church, with its internal bodies, and the State with its authorities, its policies, and its structure (judicial, legislative, and executive).

However, we can point out that the Catholic Church went through the twentieth century plagued by questions posed by the modern transformations of culture and society. New family models, patterns of morality and thought, and structural arrangements for culture and economy emerged. Tensions and alliances between Catholic groups (laity and clergy) with other spheres of values and republican-state institutions, imploded the boundary between “inside” and “outside” and launched the Catholic Church into new dilemmas. Resignification processes were accentuated with the Second Vatican Ecumenical Council, which opened an extensive bed of controversies in full boil because of its repercussions inside and outside the Catholic Church. A process that, over a wide period of time, spread to several regions and countries in which the Republican State needed to break with Catholic hegemony, especially in the Ibero-Latin and European world (Zanotto and Caldeira 2014). Current organizations advocating a Catholicism and a Church focused on moral issues and culture wars (anti-abortion campaign, against gay marriage) can be seen as heirs to ancient struggles against modernity and its secular structures (Zanotto 2012). In both new and old organizations, the recruitment of new members and the use of the media was essential, as well as the strategies of visibility in the public sphere, such as campaigns, marches in the streets, political mobilizations, but, mainly, the use of official electronic channels.

From the twentieth century to the beginning of the twenty first, Catholic action oscillated between the struggle for being present in civil society and the struggle for being influential in the state apparatus and in electoral process (Salles and Mariano 2019). In the face of all this, in particular, Catholicism has been structured on three fronts that are, in a succinct way, as follows: the first front has recovered social inspirations of struggle for equality and social justice (Christian socialism, liberation theology, Basic ecclesial communities, or CEBs [Comunidades Eclesiais de Base]); the second one has reacted to socio-political-cultural changes by seeking the centralization of papal power, clericalism, and the strengthening of integralist-traditionalist-reactionary organizations (societal religious communitarianism, nostalgia for medieval imaginary) and its releasing as a spearhead in the state, electoral and public sphere; and the third front has linked to new ways of personal charismatic experimentation, provided models linked to the society of consumption and spectacle, combining two contradictory faces, an emphasis on hyperindividualization and formation of communities of life and alliance, ending in an experimentalist mystical and in a moralistic approach, allied to reactionary groups (da Silveira 2018).

The three fronts are paradigmatically represented by CCR, CEBs/Liberation Theology, and TFP (Tradition, Family, and Property), although there are more groups linked to these three fields of practices, ideas, and narratives (da Silveira 2018). Balancing on, above and among these three forces, is the official body of Brazilian Catholicism, CNBB, which brings together all the bishops and dioceses/archdioceses. The bishops reflect, among them, the tensions between these three fronts. All official groupings were historically born between the 1940s and 1960s, the decade of the Second Vatican Council (1962), a time when ecclesiology and the Catholic administrative structure underwent profound changes: redistribution of power between the hierarchy and the laity, liturgical modernization, rationalizing rituals, devotions and behaviors, the creation of new ways of internal organization. Catholic groups acted and act in the public and media space, together with the republican and state powers, in order to promote influence and favorable perspectives to their sometimes divergent conceptions of Catholicism.

Populist structures, on the right and on the left, autocracy, and authoritarianism are accentuated in Europe and the Americas in the face of fragmentation, prevalence of environmental crises, expansion of the society of consumption, disposal, and the spectacle that permeate the way of life of some groups, social classes, religions, and societies. The undermining of formal and institutional processes of identity and political-legislative-electoral deliberation expands and, through some holes, religious groups rearrange their relations with public and state space. In Brazil, financial and cultural globalization, neoliberal advancement, and political and economic deregulation, have brought to the Nation and the modern social contract, a deep crisis of legitimacy (Ghiraldelli Jr 2020; Alencar 2019). Concomitantly, the militant religion has become a political, public, social, and cultural agent, at the transnational and local levels, mixing, on the one hand, with the struggle for the recognition of identities and, on the other hand, with the aggressive reactions of contestation to the social and cultural changes (Casanova 2008).

The three great utopias of the modern world, neoliberalism, social democracy, and communism/socialism, are in a situation of impasse and crisis, as well as the representative liberal democracies and republican structures that vertebrate the current Western societies, in the center or on the outskirts (Levitsky and Ziblatt 2018; Brown 2019). Faced with this, the movement of Catholicism in the public sphere, especially in Covid-19 era, leads us to a Gramscian idea, although it is necessary to reinterpret it: “[...] each religion, including the Catholic one [...] for its efforts to remain [...] unitary, in order not to break up into national churches and social stratifications, is a [...] multiplicity of different religions, often contradictory” (Gramsci 1966, pp. 144). The Italian philosopher continues: “[...] there is a Catholicism for the peasants, a Catholicism for the petty-bourgeois and for urban workers, a Catholicism for women and a Catholicism for intellectuals, this one varied and disconnected as well”. (Gramsci 1966, pp. 144).

Finally, in the midst of this context of the emergence of religion as a political-social agent, and in the face of a pandemic crisis in full development, with no prospect of completion, internal disputes over which segment represents the most authentic and true type of “religion” become accentuated.

## CNBB^11^ and COVID-19

Appeared in the 1950s, in the midst of social and religious changes that culminated in the Second Vatican Council, the organized group of bishops intends and is taken as the official voice of the Roman Catholic Church in Brazil and as the voice of the Catholic Church of Brazil. Third conference of bishops created in the world, its administrative composition is simple: a presidency, two vice-presidencies, a general secretary, commissions,^12^ branches, and regional offices (18 units).^13^ Its internal tensions reflect the different conceptions of the Catholic religion and the roles that it should ideally fulfill in society and in the public sphere (Klaiber 1998).

We shall not approach the great importance of its long history of action, its public quarrels with the Military Dictatorship (1964–1985) and with the Brazilian State, its relations with other Catholic bodies, its famous Fraternity Campaign on the occasion of liturgical time (Lent, Holy Week), which guides all Brazilian parishes/dioceses, its struggles for human rights and in favor of native peoples (Bruneau 1985; Klaiber 1998). This paper is interested in taking its discourse before the lethal advance of COVID-19 in Brazil.

In the case of official speeches about the pandemic, the central theme of this paper, i.e., seven “official words,” as the notes/speeches of the institution and its presidency are called, have been mapped and identified. The official website portal is simple, with links leading to its internal structure, activity, news, and tabs (official word, ministries, catechesis, family, liturgy, culture and education, ecumenism, doctrine, youth, communication, mission, laity, Amazon). The viewpoints, grouped under the “Official Word” tab, are as follows:

Chart 1 Oficial notes/speeches with release (The chart has been created from the information available on the following website: https://www.cnbb.org.br/category/palavra-oficial/ Accessed 3 Jul 2020.)

**Table:** 

May 11, 2020—CNBB’s president issues a note on the discussion of MP 910 [Provisional Measure number 910] on land regularization. In the text, the archbishop of Belo Horizonte (MG), Dom Walmor Oliveira de Azevedo, reiterates the position of the Conference whose Christian duty is to defend life, especially of the poor people, and nature. Slightly mentions the pandemic health crisis. April 4, 2020—Title: “Message from the President of the National Conference of Bishops of Brazil to the workers of Brazil”. Tribute to the Workers’ Day, with reference to the health crisis and the role of the State and to Pope Francis’ speech on decent and fair work for all. April 4, 2020—Title: “In Defense of Democracy, for Justice and Peace!” The CNBB calls on society and those responsible for public authorities to unite to prevent and combat Covid-19. By means of a note, CNBB considers that this is the most serious health crisis in recent times and claims that this is a difficult moment, which calls for the exercise of solidarity and charity. April 24, 2020—Official speech by the president of CNBB on social networks: “The change in the Ministry of Justice shows political intervention in command of institutions that, within the parameters of the Federal Constitution, must and cannot fail to have autonomy and independence”. CNBB’s President Dom Walmor Oliveira de Azevedo speaks on social media. April 18, 19 and 23, 2020—Title: “In defense of life: It’s time to take care”. Calls for support against abortion. The note is a response to the fact that the SCF [Supreme Court Federal] has scheduled the trial of ADI 5581 (release of abortion in case of Zika virus). In the text, the CNBB presidency shows concern and perplexity, in this time of pandemic, with the decision of the Supreme Court Federal (SCF) to rule for the following Friday, April 24, the treatment of the Direct Action of Unconstitutionality—ADI 5581 March 15, 2020—Title: “Time of hope and solidarity”. The message calls for unrestricted observation of medical-sanitary guidelines, expresses concern about changes in services and celebrations, and asks for attention.	

The first note is issued 20 days after the registration of the first case in Brazil, it is short, with seven points. At that point in time, the world and Brazil watched with dread the advance of the pandemic in Europe, especially in Italy and Spain. Digital platforms and large communication companies in Brazil gave great prominence to restrictive measures adopted by European governments. In the most difficult period of the pandemic, European priests and bishops died because they were infected with the new coronavirus.

The first guidelines were being taken in Brazil, and in a continuous act, there was a dispute between the minister of health, some governors and mayors and the president regarding restrictive measures. One of the excerpts from the first point of the note said:

We recommend unrestricted attention and consideration to the guidance of health experts and competent authorities. The instructions on how to celebrate the faith are up to the bishops in each diocese. All standards aim to protect people, seeking to avoid contamination and preserve life.^14^

In point four, CNBB stated: “Let us take the opportunity to think about the countless other ways in which the lives of people, peoples and the planet have been attacked.”.^15^ In the remainder, the importance of technology to supply physical distance as well as the practice of charity and solidarity are approached, but following the medical-sanitary guidelines.

About a month after the first registered case of COVID-19, CNBB issued a second official note, with seven (numbered) points, in which the biggest point was to intervene in the legal debate on whether or not to allow abortion in cases of malformation of the fetus due to the Zika virus.^16^ The tone is solemn, asserting something that is not stated in the previous note, namely, all authority coming from CNBB, the voice of Catholicism in Brazilian society:

The Presidency of the Conferência Nacional dos Bispos do Brasil (CNBB), spokesman for the Catholic Church in Brazilian society in harmony with segments, institutions, men and women of good will, calls on everyone for their commitment to the defense of life, against abortion, and publicly addresses, as it does in a personal letter, to the Ministers of the Supreme Federal Court […]^17^

The second point is a criticism of the fact that the SFC (Supreme Federal Court) scheduled the trial in the midst of the pandemic crisis. Against this, CNBB states: “It worries us all and causes us perplexity, in this serious moment of sanitary struggle for life, in this time of the COVID-19 pandemic, we are challenged to care for and support many poor and impoverished people by the worsening of economic and financial crisis [...]“.^18^

At that time, COVID-19, which had arrived in Brazil through upper-middle/wealthy travelers who had visited contaminated European regions, spread widely among the popular strata, among the most precarious and miserable areas of many cities. The very restrictive measures, taken by governors and mayors, affected the economy, producing depression, poverty, and increasing misery. The federal government, which had been following a neoliberal line of action (dismantling the State of Social Welfare), took a long time to draft some kind of public policy for social and economic support.

On the other hand, President Bolsonaro has been involved in some controversies, has denied the seriousness of the pandemic and the validity of restrictive measures and taken actions to privilege religious sectors, such as the one that considered churches as essential services, that is, services that would need to be closed or stopped. In the rest of the second note, CNBB protests against abortion, citing John Paul II’s papal encyclical letter (*Evangelium Vitae*). Regarding the theme of abortion, CNBB insists: “It is not for any public authority to selectively recognize the right to life, assuring it to some and denying it to others. This discrimination is inequitable and exclusive [...].”^19^

The third point is an official speech, but only by the CNBB’s president, dealing with a specific subject: the dismissal of the ex-judge (Sérgio Moro) who, after condemning a former Brazilian president (Lula da Silva), then candidate in 2018, accepted Bolsonaro government’s invitation to be minister of justice. Dom Walmor Oliveira de Azevedo affirms, without directly naming the controversy of the resignation, that it was a political intervention in the command of the republican institutions. There is not, in this note, any reference to COVID-19.

The fourth point, is an official note, signed by every CNBB command, reasserting an earlier document, called “Pact for Life and for Brazil”, launched on April 7, signed by 156 Brazilian institutions and civil associations. The tone of the text is strong: “In the face of the most serious health crisis in recent times, with the health system already entering the collapse phase, we consider this moment very difficult, which calls for the effective exercise of solidarity and charity.”

And, in view of the pandemonium caused by the president within the serious pandemic crisis of the new coronavirus, CNBB’s note refers to the acts of government supporters who, gathered massively in front of the Presidential Palace, called for the closure of the National Congress and the Federal Supreme Court:

We see violent demonstrations against measures to prevent coronavirus with perplexity and indignation; we have heard biased statements of contempt for life by public officials about the deaths of thousands of Brazilians infected by the covid-19; we have seen events that undermine the constitutional order, with the participation of public authorities, which defend the closing of the National Congress and the Federal Supreme Court, the return of the AI-5^20^ and the return to the dark times of the dictatorship; Brazil as a whole has heard of complaints about the politicization of justice, injuring its necessary autonomy of investigation.^21^

The note goes against the presidential performance and speeches, which defend the general opening of trade, the relaxation of quarantine and restrictive measures, in short, the economy as the life of the nation, to the detriment of the poorest people, of those who are lacking in public policies:

The care for people’s health and the economy are fundamental to guarantee life in its fullness and are not opposed to each other. In the perspective of the Church’s Social Doctrine, the economy is at the service of life: “the principle of the universal destination of benefits invites us to cultivate a vision of the economy inspired by moral values that will never lose sight of either the origin or the purpose of such benefits, in order to achieve a fair and supportive world, in which the formation of wealth can assume a positive function.” (CDSI, 174).^22^

The note seeks to criticize the division between health and caring for the economy and ends with an appeal to Brazil’s patron saint, Nossa Senhora Aparecida [Our Lady of Aparecida]. Right after that, we have a fifth point, the second official speech,^23^ restricted to CNBB’s president, reinforcing the official note (the fourth point), in celebration of Workers’ Day, May 1, as well. Dom Walmor criticizes social inequality and points to the existence of a “perverse economic logic.” The speech mentions the health, social and economic crisis, recognizing the seriousness of the problems, and also states the following:

The State has a duty to give priority to Brazilian workers in vulnerable situations. It is essential that the State adopts clear policies against the spurious interests of profit and exclusive, shameful and unjust privileges. An economy that turns to integral development is urgent, preserving jobs, income and work in the city and in the countryside, especially not allowing legislation that does not favor the dismantling of peasant survival. To you, worker who is suffering the consequences of the health and political crisis: know that you are not alone. As long as it persists, God is at your side—God will always be at your side. Solidarity is the great remedy for a new, just and fraternal world. We are together on this crossing and we will come out of it stronger. Let us be guided by hope and faith.^24^

CNBB demands the State to prioritize workers in precarious situations during the pandemic, to adopt broad public policies that are contrary to privileges and “spurious profit interests.”^25^ The official speech ends with an appeal to Saint Joseph, patron of the Church and the workers, and ends with an appeal to a just, fraternal and solidary society.

Finally, the last official position on the page is a written note issued on May 11, 2020^26^ by the president of CNBB making references to Pope Francis’ Encyclical *Laudato Si′*. The presidency’s note criticizes the imminent vote, by the National Congress, of federal legislation that deals with land regularization. The argument would be the “singular context of sanitary, economic and political crisis, crossed by Brazil”^27^ and because it is “[...] a complex theme, which involves the heritage of the union, environmental issues, land grabbing and, consequently violence in the countryside, as well as diverse interests.”^28^

As one of the most powerful bodies of the Catholic Church, CNBB, which presents itself as the spokesman for Catholicism in Brazilian society, in theory, would provide the paths to be followed by other Catholic groups organized in at least two directions proposed in this paper: the reproduction or the reference to official speeches and pronouncements or the production of proper positions, but inspired by the general guidelines offered by the association of Brazilian bishops.

However, the most conservative and the most reactionary organisms in terms of ideas and beliefs are closer to Bolsonaro’s government—a far-right government, persecuting racial, indigenous, sexual minorities, proponent of ultra-liberal policies—and come into conflict, often camouflaged and indirect, at least in the official website portals, against CNBB and Pope Francis’ guidelines.

## The Heralds of the Gospel^29^ and Covid-19

In the case of the Heralds, we see that it is one of the many conservative organizations that has emerged in the Brazilian Catholic Church throughout the twentieth and twenty first centuries. In 1960, the Brazilian Society for the Defense of Tradition, Family, and Property (TFP) was founded, which quickly expanded internationally and played a significant role in conservative Catholicism during and after the Second Vatican Council (Introvigne 2016). Its founder, Plinio Corrêa de Oliveira (1908–1995), was a prominent figure in Latin American conservative Catholicism (Introvigne 2016).

In the 1970s and 1980s, TFP was often in conflict with Brazilian liberal bishops, while proposing a definition for its internal structure, which oscillated between a group that defended a quasi-religious order and another that intended to remain as a secular lay Catholic association dedicated to political issues (Introvigne 2016). After the TFP’s founder’s death, in 1995, there was a struggle between its two main branches and the Heralds of the Gospel were born, reorganized as a religious order (Introvigne 2016).^30^

In 2001, this struggle ended and the Heralds of the Gospel were founded. The organization has grown a lot, it is now transnational (it has about 70 branches worldwide), it gathers together many priests, sisters and religious brothers, lay people, and members of the hierarchy distributed in houses and temples, in which they organize internal activities, aimed only at initiated members, and external activities, aimed at the general public.

The Heralds have the Brazilian middle and upper classes as its predominant audience, and are favorable to the defense of Catholic orthodoxy, being guided by an extreme doctrinal and moral conservatism. Catholic canon law records these associations in a variety of ways, including personal prelates and international associations of Pontifical Law followers. The complex discussions about the administrative and organizational structures of the Catholic Church at its headquarters, Holy See, and in countries and regions, are beyond the scope of this paper, but it is interesting to realize that these ecclesiastical figures are derived from the reforms of the Second Vatican Council, welcomed by the current Code of Canon Law.

Pope Francis’ pontificate has led TFP and the Heralds to some deep discomfort. The papal agenda has changed, after more than 30 years aligned with the right-wing political and economic regimes and conservatism and cultural-moral wars (battles against homosexuality, gay marriage, abortion, gender). The founders of these organizations followed an increasingly critical direction to the Vatican, and, consequently, to the organizations that follow the agenda proposed by Pope Francis.

Within a strong recovering of medieval imaginary, though romanticized and idealized, things like the rosary, the Saint James cross, the Gothic style, the strict order and discipline (temporal, behavioral, physical, spiritual), a traditional education centered on an orthodox-conservative Catholicism, with little openness to humanities and social science and philosophy (except Christian philosophy of an orthodox nature) and the idea that they are an army of soldiers and soldiers of Christ in defense of the true Catholic faith, are exalted. In 2019, accusations by former members of the Heralds exploded over sexual abuse, abuse of conscience and power, and irregular exorcism practices and the order was investigated by the state judiciary and underwent papal intervention, which resisted at first, but gave in later.^31^

The public performance of the Heralds is more spectacular, strident, with pomp, specific garments and well-defined Catholic symbols, which resort to the imagery of the High Middle Ages (fifth to eleventh centuries). The coronavirus pandemic, however, and legal provisions, have led the ultra-conservative religious order to avoid public appearances. In their social networks, the Heralds are proud to say that it was the first international association of Pontifical Law followers to be constituted by the Holy See in the third millennium on February 22, 2001. The official website portal^32^ has a serious, solemn, hierarchical design and is divided into the following tabs: who they are, videos, news, articles, donations, and “more.”

In the most central part, there are no official notes from the organization on the pandemic, nor are the CNBB’s notes echoed, but there is a large figure of Our Lady of Fatima among flowers and, within it, a link entitled “Consecrate yourself to Our Lady”. Upon clicking, a short video opens, presented by a priest from the organization dressed in a brown tunic and the cross of Saint James, half in white, half in red. It is a free online course, dedicated to the Virgin of Fatima, started in May and with new classes scheduled for July and August 2020. There is no reference to Covid-19 or its terrible health, social, and economic effects. One of the most important newspapers of capitalism, *The Economist*, has recently published a strong editorial note^33^: it recognizes not only the extreme severity of the new coronavirus pandemic in the USA and Latin America but also that the worst is yet to come and that social and economic problems will remain for long time.

On the Heralds’ website portal, the reference to the pandemic appears in a link entitled “Quarantine, faith and charity” but it is indirect and allusive, appearing only in the title web address.^34^ The link leads to a video (of 1 min) and a release, made by a priest from the organization. It is about an invitation to a campaign to collect “food and basic hygiene items throughout Brazil to be distributed to needy people through philanthropic entities, previously contacted, prioritizing those selected by the Social Assistance Secretariats and by the Diocesan Caritas.”^35^ No words are said about medical care, WHO or CNBB regulations and recommendations, and there is also no official position from that Catholic organization.

On the other hand, the new coronavirus is only mentioned in the “News” tab, but there is no reference to official notes or news from the official organization of Catholicism in Brazilian society, no positive references to WHO, no scientific magazine links about the COVID-19, and no indications for protection and health care. The collection on the website portal identified approximately 1000 links with the theme “coronavirus,” in general news, some videos from its Web-TV.^36^ However, no link leads to references favorable to WHO, to health measures, to CNBB notes, or to papal speeches that criticize socioeconomic inequalities, capitalism, racism, selfishness, the destruction of the environment, and indigenous peoples.

Most of the news, except for Web-TV videos, are reproductions, with little change, of news hosted on another website, called Gaudium Press, which presents itself as the first Catholic news agency in Brazil: “Constituted by laymen, consecrated, professionals and volunteers, Gaudium Press is a news agency that aims to spread quickly and effectively the most important events of the Catholic Church. It also broadcasts relevant articles on Catholic formation, always seeking to inform in a transparent, serious and objective way.”^37^

A first, attention to the content allows to classify this news agency as linked to a conservative-reactionary conception of Catholicism, although with some reference to the new coronavirus, sometimes neutral, without going into details. The texts inside the “news” tab of the Heralds’ website portal can be classified into exhortations to prayer, examples of sanctity, prayer for the discovery of Brazil, denunciations of abortion, news of reopening of sanctuaries, churches and holy paths (Way of Saint James, churches in England, reopening of museums and pontifical villages, reference to President Donald Trump’s request for reopening of services), requests for protection, news of face-to-face celebrations—albeit with sanitary restrictions—and celebrations of saints.

The texts of the reproduced news are short, with no indication of broad academic and scientific sources and without the signature of a reporter, journalist, or any responsible person. There is no criticism against the federal government, neither direct nor indirect, there is no text favorable to restrictive health measures, and no texts that describe the importance of sanitary measures, the socioeconomic impacts on the poor, the unemployed, or the devastation of indigenous people caused by COVID-19 either.

The oldest news that involves reference to the new coronavirus appears on internal search engines with the dating April 20, 2020 and is entitled “‘Closing of churches reminds me of the time of communism’, says Romanian Bishop.”^38^ The Greek-Catholic church bishop compares the closing of churches due to the pandemic to the closing that occurred in communist dictatorships in Eastern Europe. The text is a reproduction of a story produced by the alleged Catholic news agency, Gaudium Press, and ends like this:

But if there is one thing that is common, it is the growing desire of millions of followers from all over the world, for religious services to be resumed, even if regulations or measures must be established inside the temples to take care of their health.^39^

The second oldest news,^40^ still on April 20, 2020, is entitled: “Young Austrians to the bishops: ‘Please give us back the Mass,’” and presents a campaign for the return of Eucharistic celebrations in Austrian churches, interrupted by the new coronavirus since March 16. This campaign was imitated in Brazil by bloggers and conservative Catholic movements. The motto of the campaign was: “Dear Austrian bishops, we know that this is a very difficult period, but we only ask one thing: Give us back the Holy Mass!”^41^ This campaign has arrived in Brazil and there has been an attempt by conservative movements to put pressure on CNBB, especially through unofficial website networks, logged on to conservative Catholic lay priests. This attempt was effective not by official Catholic organizations, but by a network of priests and bloggers who, on social media, make heavy criticisms against CNBB, and praise Bolsonaro’s government. These networks have hundreds of thousands of followers and intense activity. Two of the most influential members of this conservative Catholic electronic network are Father Paulo Ricardo^42^ and Catholic blogger Bernardo Küster,^43^ who was investigated by an inquiry by the SFC about the mass production of fake news and threats to members of the judiciary.

According to the news, health issues are ignored, and there is no link that leads to scientific explanations from experts. An excerpt from Pope Francis’ words, extracted from a long homily made on April 17, 2020: “Pope Francis has recently warned about the virtual faith and a kind of ‘viralization’ of the sacraments. As he attests, ‘the Church, the Sacraments, the People of God are concrete” and cannot remain ‘virtual’.”^44^

In the way Pope Francis’ words are organized in this news, there is an ideological distortion to imply that, under the papal consent, it is necessary to reopen the churches during the pandemic, albeit carefully, vaguely mentioned and without reference to WHO or to medical experts on infectology.

As the pandemic was killing more than 30,000 Brazilians at that time, the portal posted, on June 8, 2020, the text entitled: “‘Welfare state can be as invasive as the totalitarian one’, writes President of the Bishops of France to Macron.”^45^ Despite the title, and the complete lack of knowledge of what a Welfare State is, and its public policies aimed at protecting the poor classes, minorities (sexual, ethnic) without social, political, and economic power, especially affected by the pandemic of the new coronavirus, the text says:

In response to the request of French President Emmanuel Macron, that the country’s religious leaders offer their vision and reflections on the post-coronavirus world, the president of the Conference of Bishops of France and the Archbishop of Reims, Bishop Éric de Moulins-Beaufort, wrote a Letter to the President. Addressing several topics, the 60-page document took the form of a book whose title was inspired by a phrase from Sacred Scripture: ‘Le matin, sème ton grain’ (Tomorrow, sow your seed - Letter in response to the President of the Republic’s invitation). Watch out for the state invasion. One of the themes addressed in the letter is the restrictions imposed at the time of the pandemic. Dom de Moulins-Beaufort affirms that it is not a question of complaining, but of keeping in mind that “the State always runs the risk of not seeing citizens as responsible people.”

There is a clear disjunction between the title and the content, showing a strong ideological bias on the right and reactionary side. A day later, June 9, 2020, the text, “Anchieta, hope of protection against COVID-19”^46^ is published. The news says:

In this year of 2020, the moment of sadness and apprehension through which the country to which Saint Joseph of Anchieta was an Apostle is going through coincides with his day. In fact, Brazil is experiencing the pandemic of the new coronavirus and the covid-19 is spreading all over the nation. And everything seems uncontrollable. It is not out of purpose, recalling the history of the Saint, to ask a question: If Joseph of Anchieta lived with us during this period, what would he do?^47^

The answer is given: pray, evangelize, care for the sick, do charity, and hope. Miracles and wonders of the Catholic saint who “evangelized” the Tupi-Guarani Indians and people who inhabited the Brazilian coast in the 16th and 17th centuries are dealt with. The text continues:

In the city that nowadays is called Anchieta, reports the website of the National Sanctuary of Anchieta: “in this same place, Saint Joseph of Anchieta said his own prayers, cared for and healed the sick. Here, in particular, he invoked Mary’s maternal protection against illness”.^48^

Finally, the text of the news ends with a prayer to Saint Joseph of Anchieta. Medical and health recommendations for dealing with COVID-19 are not cited, there are no references to restrictive measures, CNBB’s or WHO’s positions, no criticism is found to the federal government’s neglect of the new coronavirus pandemic, nor to its rude mistakes in leading the economic, social, and health crisis. On the other hand, the secular or non-religious international media, the largest and most respected newspapers, have highlighted these criticisms and the inadequate directions of combating the devastating impacts of the new coronavirus pandemic.^49^

## The Catholic Charismatic Renewal^50^ and Covid-19

Coming from religious experiences lived by students in 1967, the charismatic movement spread throughout the world and arrived in Brazil in 1969, brought by two American Catholic priests in the wealthiest region of Brazil, at Sao Paulo state. Shortly after, they grew up around the world and in Brazil, organized themselves in all dioceses, set up a powerful organization with thousands of groups (Carranza 2000). It has expanded a lot through large cities and middle classes, with a small penetration in rural environments and lower and working classes (Sofiati 2012).

The official website of CCR (Catholic Charismatic Renewal) is simple. The main horizontal tabs and their main subdivisions are: Home, Events (Agenda, National, State, Diocesan, National Formation School), Institutional (Who We Are, National Council, Prayer Group, Ministries, CCR States, CCR World, ICCRS, CONCCLAT, National Office, RCCBRASIL Press, Web-TV), Projects (National Headquarters, Online Courses, Communication), Spirituality and Formation (Articles, CNBB, Formation, National Intercession Network, Vatican), Collaborate, and Contact.

On the occasion of the analytical mapping, the portal’s homepage is full of announcements of saints: the apostle Thomas, St. John the Baptist, Sacred Heart of Jesus, St. Anthony, Corpus Christi, among others. There is nothing about the pandemic, its socio-health-economic impacts or references to CNBB notes. On the first page of the portal, one of the most visible links was entitled “Let us live together in fraternity among us.”^51^ It is a call for collaborations. The text says:

The purpose of Sowing Life in the Spirit is to join forces to make the Holy Spirit better known and loved. Each brother with his gift, his talent and his monthly contribution sows Life in the Spirit throughout the Brazilian nation! If you register for this project, you will contribute to RCCBRASIL, and make it possible to form Prayer Groups, social and evangelization activities in the Marajó Mission, TV Programs, Revista Renovação [Renovação Magazine], Website Portal, social networks, events in addition to helping to maintain the National Office and in the construction of the Movement’s National Headquarters. Sowing Life in the Spirit is the only national project to raise funds for RCCBRASIL activities. Moved by the Holy Spirit, the National Council for the Catholic Charismatic Renewal of Brazil has discerned that, from 2018 on, the contributions to the National Headquarters of the Movement and to the other activities of RCCBRASIL are in one project. “I will give you in abundance, do say only your yes” (Book *Vede Como Eles Se Amam* [*See How They Love Each Other*]. Prophecy given to the National Council in 2016, in the Holy Land).^52^

The text is a request to support the projects of charismatic Catholics in Brazil, such as the construction of the national headquarters, TV programs, the magazine, social media, and the website portal and its organizational structure. Despite having a link to the CNBB, the content is little and out of date, there is only one news item from April 2015.^53^ There are parts of the site that are out of date, such as the “articles” section: the last content, entitled, “O Bom pastor e as famílias ” [“The Good Shepherd and the Families”], was posted in 2016.^54^

There is no link that refers to the pandemic, the CNBB’s positions, besides no direct or indirect criticisms of the government, or even medical recommendations and references to the multiple impacts of COVID-19 on the portal either. Searches for the new coronavirus returned 16 links. On the Heralds’ page, there was a lot of news reproduced from an agency, but on the CCR page, there is little, which allowed us to build a specific picture:

Chart 2 Texts about pandemic/coronavirus(Available via https://rccbrasil.org.br/espiritualidade-e-formacao/pesquisar.html?searchword=pandemia&ordering=&searchphrase=all. Accessed 5 Jul 2020.)

**Table:** 

Title/date	Release
Reajustando a armadura para o combate! [Readjusting the armor for combat!] (Rede Nacional de Intercessão/Rede Nacional de Intercessão)—July 1, 2020	With biblical quotes, the text talks about spiritual combat and the need to pray or intercede. Fifteen permanent intentions to pray are listed
2. Unidade da Igreja: Dom do Espírito Santo [Church Unity: Gift of the Holy Spirit]—June 1, 2020	It talks about Pope Francis’ homily on Pentecost day. Mentions the pandemic, criticizes narcissism, pessimism, saying that, for God, everyone is a child, besides right or left
3. Examinai tudo: abraçai o que é bom [Examine everything: embrace what is good]—May 6, 2020	With biblical quotes, the text invites to intercessory prayer. Fifteen permanent intentions to pray are listed
4. Mês Mariano: Recomendações do Papa Francisco [Month of Mary: Pope Francis’ Recommendations]—April 27, 2020	The text contains prayers to the Virgin Mary
5. Catequese: A luz da fé revelada na criação [Catechesis: The light of faith revealed in creation]—April 22, 2020	World Earth Day
6. Como viver bem o período de quarentena em família? [How to live the family quarantine period well?]—April 14, 2020	There is a speech by Pope John Paul II, bringing tips for staying in quarantine, as a family: praying a rosary, recreational activities, etc.
7. Celebrando a Paixão de Cristo [Celebrating the Passion of Christ]—April 9, 2020	Good Friday. It recommends attending the rituals by means of communication, fasting, prayer and veneration of the crucifix
8. Catequese: A Cruz de Cristo é a resposta para a dor humana [Catechesis: The Cross of Christ is the answer to human pain]—April 8, 2020	A short text, mentioning the pandemic, but focusing on prayers
9. Família carismática unida em oração pelo Mundo [Charismatic family united in prayer for the World]—April 7, 2020	It is about the National Day of Intercession with a schedule of prayers to be said and types of prayer
10. O que é comunhão espiritual? [What is spiritual communion?]—April 6, 2020	In view of the impossibility of physical meeting, spiritual communion is proposed
11. Jesus quer entrar no seu coração! [Jesus wants to enter your heart!]—April 3, 2020	Palm Sunday
12. A vida no Espírito para resgatar a identidade [Life in the Spirit to rescue identity] April 3, 2020	Invitation of the National Intercession Network. It is also asked for an end to the pandemic coronavirus in all countries
13. Decreto da Congregação para o Culto Divino sobre a Semana Santa [Decree of the Congregation for Divine Worship on Holy Week]—March 30, 2020	Mention of an ecclesial decree recommending that physical ceremonies be performed due to the Covid-19 pandemic
14. RCCBRASIL programa momentos de espiritualidade nas Redes Sociais [RCCBRASIL programs moments of spirituality on Social Networks]—24/03/2020	It recognizes routine changes due to the Covid-19 pandemic and encourages the use of the movement’s social media
15. Como estender seu Grupo de Oração no ambiente virtual [How to extend your prayer group in the virtual environment]—19/03/2020	It is recommended to use social networks and applications such as zoom, hangouts for charismatics to meet during the pandemic
16. CNBB convida brasileiros para oração do terço. [CNBB invites Brazilians to pray the Rosary] 18/03/2020	National prayer of the Rosary, coordinated by CNBB

Text 16, in Chart 2, dates from March 18, 2020 and is the first to talk about the pandemic and is entitled: “CNBB invites Brazilians to pray the rosary.”^55^ This is an invitation:

Faced with the Covid-19 pandemic, the new coronavirus, the National Conference of Bishops of Brazil (CNBB), in communion with Pope Francis in a commitment to intensify prayers in this period, joins Brazil calling on everyone for a moment of prayer to be held today, March 18, Wednesday, at 3:30 pm. On the occasion, the presidency of the CNBB, together with religious and lay guests, will pray the Rosary of Hope and Solidarity, which will be broadcast on all Catholic-inspired televisions in the country, radio stations, through the Conference page on Facebook and Youtube. The initiative, especially in delicate and difficult moments like this, seeks to raise hearts to the God of Life, in accepting his Word, strengthening faith, hope and unity. “Aware that restrictions on living together will not last forever, let us learn to value fraternity, making us even more desirous that, after the pandemic, we can be together, celebrating life, health, harmony and peace” (excerpt from the note “Times of Hope and Solidarity” from CNBB).^56^

The text mentions the pandemic in a superficial way, talks about delicate times, indicates the spiritual search as more important and ends with the reproduction of part of the first official note of the CNBB (health restrictions would be temporary).

The fourth text talks about quarantine, restrictions, suggesting activities, but it does not talk about WHO measures, political-social problems, the Ministry of Health, health measures, the impacts of COVID-19, and the dead ones. An excerpt shows how far the charismatic discourse goes in a parallel reality: “A magical world can be created without leaving the living room rug, with just a dose of creativity.” And further:

It is essential to live this time in the spiritual vision, because the Lord slowed the world down for a purpose, maybe it was just to make us look inside ourselves, at those we love, at everything that really matters in our lives and tell our God: “Take me where men need your Word, even if it is inside my own home.”

Text nine states:

In view of the increase in cases of Covid-19 (Coronavirus) in all countries, in the face of this pandemic, our attitude should not be one of fanfare, but of faith and trust in God. Therefore, we are committed to redouble prayers and obey local pastoral guidelines.^57^

Text 11 goes back to spiritual search, but it is a war:

I am not afraid to say: we are in a war. A war that is not just epidemiological. It is also spiritual. The Enemy wants to take the people of God out of Mass, but his shot backfired. There has never been so much Mass offered by the media. Cell phones have reduced the distance between shepherds and sheeps, between temples and baptized people. The Enemy wants to get the people out of the church, but the Church is increasingly present in people’s hearts. In this year of 2020 there is only one possibility: Jesus entering the Jerusalem of our hearts, and walking the streets of our souls, since there will be no processions on the streets of our cities.^58^

Text 1 is the most recent published during the research. It is an invitation to pray and be part of the “National Intercession Network”, and 15 permanent points are listed, repeated every month, for charismatics to pray, but the pandemic is not mentioned:

1. For the Holy Church, for the Holy Father, Pope Francis, for the Bishops, for the Priests, Deacons, Religious and Seminarians; 2. For all vocations, so that God’s call may be taken up with love and fidelity; 3. For the members of the International Service for Catholic Charismatic Renewal; 4. For the members of the National Communion Service; 5. For the President of the National Council, Vinícius Simões and his family, and all members of the National Council; 6. For the meetings of the State and Diocesan Councils; 7. For all the Prayer Groups in Brazil; 8. For all the Ministries of the CCR at the national, state, diocesan and prayer group levels; 9. For the spiritual and financial needs of the diocesan, state and national offices of the CCR; 10. For the mission house of RCCBRASIL in Marajó and for the missionaries; 11. For the construction of the National Headquarters of CCR in Brazil and its collaborators; 12. For the events of evangelization of the CCR in Brazil; 13. Due to the political, economic and moral situation in our country; 14. To stop violence in Brazil and in the world; 15. For the eradication of the viruses that cause Yellow Fever, Dengue, Zika and Chikungunya.

The order of the permanent prayer points, their content, and absences are expressive and show symptoms of semantic alienation. It starts with the Church and its leaders (pope, bishops, priests, and seminarians), but does not mention CNBB. Then it is asked for vocations in general (laity is not mentioned), and most of the intentions are for the movement itself and its structures (items 3, 4, 5, 6,7,9, 10, 11, and 12).

In the end, there is a general discussion of the Brazilian situation, its violence and four diseases and viruses (Yellow Fever, Dengue, Zika and Chikungunya) except SARS-CoV-2. Prayers are not asked for health professionals, especially those dealing with the pandemic or political authorities, neither federal nor state.

However, COVID-19 continues to kill thousands of Brazilians, the majority of whom are elderly, black, or indigenous people, residents of slums and outskirts, unemployed or with very precarious work, and homeless people as well. We barely know the size of the biological and medical, social, and economic problems that are involved, but they are many and are just beginning.^59^

Despite the enormous territorial dimensions, with 27 state capitals, many metropolises, the pandemic curve is rising in many areas and only a few have stabilized. Cities that came out of quarantine and reopened trade are being forced to close again. Finally, the text exhales a military, warlike language:

Sacred Scripture says: ‘The weapons we fight with are not carnal. They are powerful in God, capable of destroying fortifications. We annihilate all reasoning and pride that arises against the knowledge of God, and captivate every thought and reduce it to obedience to Christ’ (Second Corinthians X, 4-5). In the arsenal of the Lord we find powerful offensive and defensive weapons, without which we will not obtain victory, requiring us, though, some training in each of them, namely: The catapult is the PRAISE, our crossbow is THE NAME OF JESUS, our onager is the WORD OF GOD AND THE AUTHORITIES CONSTITUTED, our battering ram is the SPIRITUAL GIFTS, our testudo is the HOLINESS OF LIFE, our vine, is the BLOOD OF JESUS and our arrows, are the PRAYER IN THE SPIRIT. The conviction that the Lord Himself marches before us must fill us with strength and courage, because He empowers us, gives us strategies, provides us with necessary spiritual weapons and fights on our behalf. ‘The Lord will fight for you; as for you, you will have nothing to do.’ (Exodus XIV, 14)

The description of medieval or tribal weapons of war that are translated by spiritual gifts, holy life (without sinning) call our attention, as well as prayer written in capital letters. The onager is called the Word of God and constituted authorities, very broad terms that can include any interpretation, for example, obedience to the Federal Government or to state governments, which are in conflict.

It is interesting to notice that from March to July, the news neglect to mention the COVID-19 pandemic explicitly, reproducing only the invitation to prayer, made by CNBB, but not its most critical notes, not mentioning the dead people, their suffering, the health measures, social problems, or political conflicts.

Finally, we have the only official public positioning, on July 2, 2020, signed by the president of CCR in Brazil. It is entitled: “Letter to charismatics. ‘Revive the flame of the gift of God’ (Second Timothy I, 6).”^60^ In this text, we see a call to revive spirituality, not to take your eyes off Jesus, to overcome the seductions of the world, to have perseverance in faith, to know how to distinguish the voice of the true shepherd (Jesus) so as not to be confused with similar voices and to resort to the intercession of Our Lady of Pentecost. No mention is made of the Covid-19 pandemic and its multiple psychological, social, economic impacts, CNBB, health recommendations, WHO, science or academic research, or even political issues. The text ends with an exclamation in Latin: Veni Sancte Spiritus! (Come Holy Spirit!). How these issues remain within a broader picture is the next step in this paper.

## The Public Sphere and Catholicisms

From what can be seen, since the 1990s, there has been an expansion of the political arena with the growing participation of Evangelicals and the emergence of new actors in civil and political societies, especially the identity movements (Machado 2012). Disputes have intensified in public arenas in Brazil among groups polarized between those (feminists, homosexuals, intellectuals, journalists, educators, lawyers, artists, state managers, among others) that defend the establishment of public policies regarding health, education, scientific research, and legal order that would guarantee minorities human, sexual, and reproductive rights without religious interference versus conservative religious groups, which, for reasons of creed and morals, would maintain their actions within the legal-political expedients of the republican framework to assert their proposals for dismantling such initiatives from secular forces (Camurça 2017).

The Heralds of the Gospel and the CCR are the spearhead of a conservative Catholic reaffirmation that aligns with conservative Evangelical sectors, acting in the National Congress, in relation to moral issues, for instance, against bills that aim to incorporate as civil rights issues such as same-sex marriage and legalization of abortion (Burity 2006). These religious groups are heterogeneous, but adhere to a spiritual-conservative discourse, which implies a consensual aptitude that enables the convergence between evangelical denominations and expressive segments of the Catholic Church, bishops and conservative clergy, charismatic movements, and traditionalist communities (Datta 2018).

An agenda of conservative conceptions of the family and society stands out, prevailing the idea of middle-class Christian supremacy (Pierucci). It is worth mentioning that in the case of the presidential elections in 2018, considerable segments of the Catholic Church of CNBB and of the social and youth Pastorals, together with minority Evangelical segments, did not express explicit support for Fernando Haddad’s (PT) candidacy, but the ideas around it were supported because they defended a social agenda and that, in relation to religion, they strive for recognition and respect for religious plurality by the State (Camurça 2019).

Conservative Catholic activists have established rapprochements with similar Evangelical groups. We understand that a significant portion of these Catholics make up the broader social process called, in public debate, conservative wave, which articulates, at different levels, at least four lines of social forces: economic liberalism, moral regulation, and social and punitive intolerance (Almeida 2019). However, it shall be necessary to problematize the concept of “wave,” as this conceptual construction undermines structures of long religious duration. In societies with extreme, profound and historical, social, cultural, racial, economic and political inequality, such as the Brazilian one, long-term structures tend to be strongly conservative and, a good part of them, virulently reactionary.

The conservative and reactionary Christian segments have acted to “restore” the supposed traditional moral and social order that, in their view, would be under attack by “evil forces.” The anti-gender, anti-science, and anti-pluralist struggles reproduce moral repertoires and political battles of the Christian right and also of the Vatican, such as the notion of “gender ideology,” an ideological weapon that has become ubiquitous in parliamentary elections and disputes in the region (Silveira 2019). In addition, recent political polarization has helped to consolidate a Christian right, divided into more or less fanatical groups, on social media and in the public sphere. The rejection of the PT (Partido dos Trabalhadores, in English “Workers’ Party”) and anachronistic anti-communism, have increasingly guided political positions of conservative Christian leaders and deputies. More and more, these ones began to identify themselves as conservatives, to ally themselves with right-wing groups, to attack human rights, sex education in schools, and anti-homophobic policies (Mariano and Gerardi 2019).

It is worth noting that precisely within the scope of the *Comunidade de Aliança e de Vida Canção Nova* [*Canção Nova’s Alliance and Life Community*],^61^ a powerful arm of the RCC-Brazil, there is a representative portion of the fiercely political activism of representatives of the Catholic conservative wing, including members of the episcopate, regional leaders of CNBB, and priests and lay people who joined the candidacy of Jair Bolsonaro (PSL-RJ) in the presidential elections of 2018. A significant part of these conservative Catholics was together with some Evangelical segments in the Brazilian crisis expressed and unleashed in/with the street protests of June 2013, mostly democratic, which became polarized in the 2010 and 2014 elections, and deepened with Dilma Rousseff’s impeachment (2016).

We argue, however, that the vast ideological field of reactionary Christianity and conservative Christianity, in the Catholic and Evangelical strands, can be seen, in this journey from the 2000s to the 2020s, in two segments, one more pragmatic, which has supported, for example, the left (PT/Lula/Dilma) and another, more ideological, distant from the governments on the left. However, in the 2018 elections, these two segments converged and, based on old conservative bases present in Brazilian society, they presented new ways of being present, including within social networks and media. This was reflected in a significant way in the conditions of possibility of Jair Bolsonaro’s speeches regarding morality and customs and for his enchantment experienced as an evidence in adhering to conservative discourse (Almeida 2019).

Like Evangelicals, the speeches aforementioned reinforce Bolsonaro’s role in the defense of a Brazilian Christian nation, whose ethical-moral values should guide all the society and inspire the formulation of public policies. Such religious agents recognize him as possessing the symbolic capital of “president of the Christians,” consolidating the image of Brazil as a Christian nation and reinforcing the religious character in the President’s determinations.

Based on all this configuration, the Federal Government enacted, in June 2020, a decree (n° 10,292) that places religious worship activities as essential activities during the COVID-19 pandemic. This Presidential Decree was criticized by CNBB, in a statement signed by Secretary General, Dom Joel Portella Amado, who highlights the

... guidelines issued by the competent authorities of the Ministry of Health that indicate social distance, churches, if the bishops consider it so, can remain open, however, in the way it has been done: individual prayers, online transmissions etc. There is no way to understand that the aforementioned legal instruments may force the reopening of churches, much less for the practice of any type of agglomeration.^62^

The pronouncement is still in solidarity with the religious assistance to the sick, to health professionals and people in general and, on the other hand, to follow the health rules, the basis of which is social distancing, in spite of some specifically pastoral concerns, some issues related to livelihood through temporal and charity benefits that churches need.

The Heralds of the Gospel and the CCR insist on minimizing the pandemic directly or indirectly. They do not issue official notes of any kind, they simply reproduce or produce news that does not address the impacts of Covid-19, ignoring the official positions of CNBB, and not criticizing the evident health and social flaws of the federal government. In a way, these entities show an interest in participating in disputes in the Brazilian religious field, especially regarding the new boards and temporalities of contemporary political dynamics, marked by disorientation and unpredictability, gaining new chapters with the ideological polarization instituted in epidemic times (Almeida 2019).

Through the Episcopal Pastoral Council [Conselho Episcopal Pastoral—CONSEP], CNBB has reassured, in a note, its commitment to the *Pact for Life and for Brazil*, released on April 7, 2020, to the World Health Day, initially signed by six civil society institutions^63^ and subsequently by more than 150 entities. This manifesto recognizes the seriousness of the sanitary, economic, social, and political crises, highlighting the need to exercise citizenship guided by the principles of solidarity and human dignity, especially by government officials and representatives of the people, as well as the search for joint solutions for common welfare, particularly of the poorest and most vulnerable people. The document calls for the union of society, confirms the importance of social isolation to slow the transmission of the virus and its contagion, preserving the action capacity of health systems, and also asks for the implementation of public social protection policies, including measures for economic replacement for the most vulnerable segments:

It is time for the lucid chorus to enter the scene in Brazil, making the choice for scientific, political and social models that put the world and our society in a really new time. Our civil society expects, and has the right to demand, that the Federal Government be the promoter of this dialog, presiding over the process of great and urgent changes in harmony with the powers of the Republic, overcoming the folly of provocations and personalisms, to stick to the principles and values enshrined in the 1988 Constitution. It is worth remembering that the arduous task of fighting the pandemic is everyone’s duty, with the participation of all – in the case of the Federal Government, in articulated cooperation with the governments of States and Municipalities and in close connection with our institutions. The time is serious and calls for ethical, bold, and humanistic leadership, which echoes a pact signed by the whole of society, as a commitment and compass for overcoming the current crisis.^64^

The letter rejects the speeches criticizing the effectiveness of this strategy, putting the health and survival of the Brazilian people at risk. It defends support for the guidelines of national health organizations, such as the Ministry of Health, and international ones, starting with the World Health Organization—WHO. It underscores the importance of the Unified Health System [Sistema Único de Saúde—SUS], once again confirmed, with its thousands of agents risking their own lives on the front lines of the fight against the pandemic.

Groups linked to the episcopate defend the President’s impeachment. Bishop of Vacaria (RS), Dom Silvio Guterres Dutra, for example, accused the president of spreading “division and confusion” and rebuked the acts with a religious tone in the vicinity of power. “Unfortunately, some of Bolsonaro’s so-called ‘supporters’ have starred in real pharisaic scenes of public prayers, of blatant false veneration for the sacred images of Our Lady of Fatima and Jesus of Divine Mercy in an attempt to give legitimacy to their thoughts.”^65^

On the other hand, the president called a national day of fasting, on the day of Christian Easter (April 7, 2020). He prayed kneeling in the lobby of the Palácio da Alvorada, raised an image of Merciful Jesus on the Palácio do Planalto’s ramp, and promoted the aforementioned videoconference with religious leaders at Easter, with Evangelicals and Catholics, without authorization or prior communication to CNBB, which places itself and is seen, by broad social, religious and non-religious groups, as a spokesman for Catholicism in Brazilian society.

## Concluding Remarks

What can be seen in the pandemic, which in countries like Brazil is expected to be long, is the worsening of tensions between reactionary and conservative organizations (Heralds and RCC) and CNBB, seen to a large extent as the Catholic voice par excellence, and legitimacy in Brazilian society.

The analysis of website portals shows a divorce between conservative and reactionary organizations, aligned on a common social-media terrain, and the pastoral lines of the largest Brazilian Catholic body. The Heralds and the CCR ideologically commune with the government, by not criticizing the serious problems arising from the president’s action against more effective sanitary and health measures as well as social protection and support, especially for groups such as indigenous peoples. The CNBB, however, has continued its role as a critic of the State’s actions and the defense of values of equality and social justice. Perhaps the only point of convergence between these three organizations is the position against abortion, which is a polemical and controversial issue.

The dispute over which Catholic groups would have the legitimacy to position themselves in the Brazilian public sphere repositions the actors in opposite fields. The specialized agents and spokespersons—the CNBB, Heralds of the Gospel and CCR, invested with power, show themselves capable of responding, more or less, through practices and speeches, to their own needs and to those belonging to certain social groups, within and outside the institution.

In the full term of the coronavirus pandemic, the ways of adaptation, accommodation, and the relationship between the different agents and spokespersons of the main institutional segments, not to mention the direction of power relations in the Brazilian political field, still seem undetermined in the complex Catholic field. However, the radicalization of the discourse supporting the government seems to find limits within the institution, both in terms of theological counterpoint and in political terms, issued by religious congregations and CNBB.

## Footnotes

1 For methodological reasons, the understanding of the virus shall not be deepened here, but it shall be understood as a political and social entity, because, even though it consists of biological mechanisms (a filament of RNA (Ribonucleic Acid) that coils in the membranes of human cells, introducing its genetic material and forces them to produce copies of the infesting virus), the virus is in fact the result of human policies against nature and policies to promote the life of the nation and the (neoliberal) economy to the detriment of the poorest and the unemployed people’s lives, or those with precarious work, as well as ethnic, sexual, religious minorities, etc., who have no social and economic power. There is no biological virus because every virus, when it enters human history, is already political, social, economic, cultural, and religious.

2 Available via https://coronavirus.saude.gov.br/sobre-a-doenca#o-que-e-covid. Accessed 12 Jun 2020.

3 Within the limits of this paper, we shall define neoliberalism as the doctrine and political-economic practice that fights for the withdrawal of the State from broad sectors of society, for the privatization of public services, for the total and absolute freedom of the individual, even in the face of restrictive health rules, such as wearing masks. For further reading, see Brown (2019) and Ghiraldelli (2020).

4 Brazilian President’s middle name is Messias (Jair Messias Bolsonaro), the Portuguese for Messiah. There is a kind of pun here between his middle name and the person of Jesus of Nazareth, believed to be the Messiah by all those who follow Christianity, just like President Bolsonaro. This link shows these and other phrases spoken by President Bolsonaro: Available via https://g1.globo.com/politica/noticia/2020/04/30/veja-frases-de-bolsonaro-durante-a-pandemia -do-novo-coronavirus.ghtml Accessed 3 jul. 2020.

5 At the moment this paper was being written, July 4, 2020, the state health departments (27) accounted for 1,543,341 infected people and 63,254 deaths. According to federal government accounts, there are 1,539,081 infected people and 63,174 deaths. Available via: https://noticias.uol.com.br/saude/ultimas-noticias/redacao/2020/07/03/coronavirus-covid-19-casos-mortes-3-julho.htm Accessed 2 Jul 2020.

6 The percentages refer to the national population.

7 On May 9, 2020, one of the most prestigious international scientific journals on infectious diseases, *The Lancet*, published an extremely critical editorial entitled: *COVID-19 in Brazil: “So what?”* (Available via https://www.thelancet.com/journals/lancet/article/PIIS0140-6736(20)31095-3/fulltext#articleInformation Accessed 03 Jul 2020). On June 11, 2020, this journal published a new editorial entitled “*Political casualties of the COVID-19 pandemic*.” (Available via: https://www.thelancet.com/journals/laninf/article/PIIS1473-3099(20)30496-5/fulltext Accessed 03 Jul 2020).

8 See the report: *Le Monde*. Au Brésil, des évangéliques nient la dangerosité du coronavirus. (Available via: https://marketingeditorare.wixsite.com/ebooks. Accessed 21 Jun. 2020).

9 In 2010, 64% said they were Catholics. Evangelicals, grouped under different denominations, reached 22% of the Brazilian population, and within those 22%, more than 15% are Pentecostal and Neopentecostal followers. However, it is necessary to pay attention to regional disparities: there are more Catholic regions and less Catholic regions, states, and cities, as well as more Evangelical or less Evangelical ones.

10 On the first confirmed case in Brazil: Available via https://www.saude.gov.br/noticias/agencia-saude/46435-brasil-confirma-primeiro-caso-de-novo-coronavirus Accessed 2 Jul. 2020.

11 The official page, available via https://www.cnbb.org.br/ Accessed 4 Jul 2020.

12 The most famous, including the confrontation they have with governments, since the military government (1965–1985), up to the current one (2018–2022), are CIMI (Conselho Indigenista Missionário [IMC—Indigenist Missionary Council]); CPT (Comissão Pastoral da Terra [LPC—Land Pastoral Commitee]); CEFEP (Centro Nacional de Fé e Política [NCFP—National Centre for Faith and Politics]); CBJP (Comissão Brasileira de Justiça e Paz [BCJP—Brazilian Committee for Justice and Peace]).

13 To get an inside look at its story, see: https://www.cnbb.org.br/a-estrutura-administrativa-e-pastoral-da-cnbb-tem-uma-matriz-e-18-unidades-regionais/. Accessed 3 Jul 2020.

14 Available via https://www.cnbb.org.br/cnbb-emite-mensagem-na-qual-pede-observacao-irrestrita-as-orientacoes-medico-sanitarias/ Accessed 3 Jul 2020.

15 Idem, ibidem.

16 It was a judgment made by the SFC (Supreme Federal Court) on the constitutionality of abortion requests from mothers who have generated fetuses with severe disabling deformities for life. The Zika-Virus is another large family of viruses that started to circulate in the world, and in Brazil, from Africa. It is related to poor conditions of water and sewage treatment, and absolute poverty.

17 Available via https://www.cnbb.org.br/a-presidencia-da-cnbb-publica-a-nota-em-defesa-da-vida-e-tempo-de-cuidar-para-pedir-a-todos-o-empenho-pela-o-defesa-da-vida/ Accessed 3 Jul 2020.

18 Available via https://www.cnbb.org.br/a-presidencia-da-cnbb-publica-a-nota-em-defesa-da-vida-e-tempo-de-cuidar-para-pedir-a-todos-o-empenho-pela-o-defesa-da-vida/ Accessed 3 Jul 2020.

19 Idem, ibidem.

20 Reference to the most authoritarian, hard and dictatorial act taken by the Brazilian military regime in 1968.

21 Available via https://www.cnbb.org.br/cnbb-conclama-a-sociedade-e-os-responsaveis-pelos-poderes-publicos-a-se-unirem-a-prevencao-e-o-combate-a-covid-19/. Accessed 4 Jul 2020.

22 Idem, ibidem.

23 Recorded on video on CNBB’s YouTube channel. Available via https://www.youtube.com/watch?v=3bSg3w6GIqQ&feature=youtu.be. Accessed 4 Jul 2020.

24 Available via https://www.cnbb.org.br/mensagem-do-presidente-da-cnbb-aos-trabalhadores-as-do-brasil-o-momento-e-de-uniao-de-esforcos-publico-e-privado-para-que-ninguem-seja-deixado-para-tras/ Accessed 4 Jul 2020.

25 Idem, ibidem.

26 Available via https://www.cnbb.org.br/presidente-da-cnbb-emite-nota-sobre-a-discussao-da-mp-910-da-regularizacao-fundiaria/ Accessed 4 Jul 2020.

27 Idem, ibidem.

28 Idem, ibidem.

29 Official website: Available via https://www.arautos.org/ Accessed 4 Jul 2020.

30 See the founder’s biography, Brazilian Monsignor João Scognamiglio Clá Dias. Available via https://www.arautos.org/secoes/artigos/especiais/biografia-monsenhor-joao-cla-dias-260690 Accessed 4 Jul 2020.

31 Available via https://www.vaticannews.va/en/vatican-city/news/2019-09/pope-approves-commissioner-heralds-gospel.html Acccessed 4 Jul 2020.

32 Available via: https://www.arautos.org/ Accessed 4 Jul 2020.

33 Available via https://www.economist.com/leaders/2020/07/04/covid-19-is-here-to-stay-people-will-have-to-adapt Accessed 4 Jul 2020.

34 Available via: https://www.arautos.org/quarentena-caridade-ajude-covid19 Accessed 4 Jul 2020.

35 Idem, ibidem.

36 Available via http://www.arautos.org/tv/home/index/id_video/234+/5/3/page/15/2.html Accessed 4 Jul 2020.

37 Available via https://gaudiumpress.org/ Accessed 4 Jul 2020. It is not known, in fact, who is behind this agency, whom it obeys, what are the criteria for publishing news and who are responsible for the content produced. There is no record of professional journalists. The website presents news in four languages: Portuguese, English, Italian, and Spanish. They have a WhatsApp number available to anyone who wants to receive their news. There is a Facebook page, but it is not possible to identify who is responsible for it, although it is known that there are six Brazilian and one Mexican (anonymous) administrator. This Facebook page was opened in 2011 under the name “Gaudium Press” and in 2020 changed its name to “Gaudium Press—Catholic News Agency—the Church in Brazil and in the world”. It has 11,000 followers, many of them priests. On Instagram, Gaudium Press has few followers (220) and on Twitter, it has 6000 followers.

38 Available via: https://www.arautos.org/secoes/noticias/noticia/fechamento-de-igrejas-me-lembra-da-epoca-do-comunismo-diz-bispo-romeno-287672 Accessed 4 Jul 2020.

39 Idem, ibidem.

40 Available via https://www.arautos.org/secoes/noticias/noticia/jovens-austriacos-aos-bispos-por-favor-devolvam-nos-a-missa-287666 Accessed 4 Jul 2020.

41 Idem, ibidem.

42 To know more about this Father, see Silveira (2019).

43 Available via https://brasil.elpais.com/brasil/2020-05-27/policia-federal-mira-aliados-de-bolsonaro-em-investigacao-sobre-noticias-falsas.html Accessed 4 Jul 2020. This blogger was condemned by justice for slandering one of the founders of Liberation Theology in Brazil, Leonardo Boff. Available via https://politica.estadao.com.br/noticias/geral, justica-mantem-condenacao-a-youtuber-bolsonarista-que-atacou-leonardo-boff,70,003,294,747. Accessed 4 Jul 2020.

44 Idem, ibidem.

45 Available via: https://www.arautos.org/secoes/noticias/noticia/estado-de-bem-estar-pode-ser-tao-invasivo-quanto-o-totalitario-escreve-presidente-dos-bispos-da-franca-a-macron-290263. Accessed 4 Jul 2020.

46 Available via: https://www.arautos.org/secoes/noticias/noticia/anchieta-esperanca-de-protecao-contra-a-covid-19-290388. Accessed 4 Jul 2020.

47 Idem, ibidem.

48 Available via https://www.arautos.org/secoes/noticias/noticia/anchieta-esperanca-de-protecao-contra-a-covid-19-290388. Accessed 4 Jul 2020.

49 For example, a text from *The New York Times*, “Bolsonaro Is Bizarre. But He Knows What He’s Doing. Brazil’s president does not need to centralize power to get his way”. Available via: https://www.nytimes.com/2020/06/03/opinion/bolsonaro-brazil-coronavirus.html?searchResultPosition=2. Accessed 4 Jul 2020.

50 Official website available via https://www.rccbrasil.org.br/portal/. Accessed 5 Jul 2020.

51 Available via https://www.rccbrasil.org.br/projetos/eu-colaboro/. Accessed 5 jul. 2020.

52 Idem, ibidem.

53 vailable via https://www.rccbrasil.org.br/espiritualidade-e-formacao/cnbb.html. Accessed 5 Jul 2020.

54 Available via https://rccbrasil.org.br/espiritualidade-e-formacao/artigos.html. Accessed 5 Jul 2020.

55 Available via https://rccbrasil.org.br/espiritualidade-e-formacao/mais-lidas-cnbb/2197-cnbb-convida-brasileiros-para-oracao-do-terco.html. Accessed 5 Jul 2020.

56 Idem, ibidem.

57 Available via https://rccbrasil.org.br/espiritualidade-e-formacao/mais-lidas-intercessao/2213-familia-carismatica-unida-em-oracao-pelo-mundo.html Accessed 5 Jul 2020.

58 Available via https://rccbrasil.org.br/espiritualidade-e-formacao/mais-lidas-artigos/2209-domingo-de-ramos-jesus-quer-entrar-no-seu-coracao.html Accessed 5 Jul 2020.

59 Available via https://www.nytimes.com/2020/07/04/health/239-experts-with-one-big-claim-the-coronavirus-is-airborne.html?searchResultPosition=2 Accessed 4 Jul 2020.

60 Available via https://www.rccbrasil.org.br/institucional/index.php/artigos/1422-qreaviva-a-chama-do-dom-de-deusq-cf-ii-tm-1-6 Accessed 4 Jul 2020.

61 It is one of the most powerful and rich expressions of charismatic Catholicism. Founded in 1978 by Father Jonas Abib and some young people, in a city close to Sao Paulo. It has become a large organization, with branches throughout Brazil, the USA, and Europe, owner of a communication network with TV, Radio, Website Portal, a college, and a large building complex at its own headquarters: temples, places of worship, large trade in own brands, etc. (Silveira 2016).

62 Available via https://g1.globo.com/politica/noticia/2020/03/26/lideres-religiosos-comentam-decreto-de-bolsonaro-que-torna-atividades-religiosas-essenciais-em-meio-ao-coronavirus.ghtml Accessed 5 Jul 2020.

63 The document is signed by: Dom Walmor Oliveira de Azevedo, president of the Conferência Nacional dos Bispos do Brasil—CNBB, Felipe Santa Cruz, president of the Order of Attorneys of Brazil—OAB, José Carlos Dias, president of the Commission on Defense of Human Rights [Comissão de Defesa dos Direitos Humanos], Dom Paulo Evaristo Arns—Arns Commission, Luiz Davidovich, president of Brazilian Academy of Sciences [Academia Brasileira de Ciências - ABC], Paulo Jeronimo de Sousa, president of the Brazilian Press Association [Associação Brasileira de Imprensa—ABI], Ildeu de Castro Moreira, president of the Brazilian Society for the Advancement of Science [Sociedade Brasileira para o Progresso da Ciência—SBPC].

64 Available via https://www.cnbb.org.br/wp-content/uploads/sites/32/2020/04/pacto-pela-vida-e-pelo-brasil-.pdf. Accessed 5 Jul 2020.

65 Available via https://politica.estadao.com.br/noticias/geral,divisao-na-igreja-abre-caminho-para-bolsonaro-negociar-com-veiculos-catolicos-simpaticos-ao-governo, 70003326557. Accessed 5 Jul 2020.

## References

[CR1] Alencar, GF (2019) Um país laico com um governo terrivelmente cristão? Interações, Belo Horizonte, Brasil, 14/25, pp. 13–28, jan./jun.

[CR2] Almeida, R (2019) Bolsonaro Presidente: Conservadorismo, evangelismo e a crise brasileira. Novos Estudos Cebrap. São Paulo, 38/1, pp 185–213, jan.–abr

[CR3] Bandeira, O and Carranza, B (2020) Só o Brasil Cristão Salva da Covid-19. Boletim n.33, Ciências Sociais e Coronavírus, 5 may 2020

[CR4] Brown W (2019). In the ruins of neoliberalism. The Rise of Antidemocratic Politics in the West.

[CR5] Bruneau T (1985). Church and politics in Brazil: the genesis of change. J Lat Am Stud.

[CR6] Burity J (2006). Redes, parcerias e participação religiosa nas políticas sociais no Brasil.

[CR7] Camurça M (2017). A Questão da laicidade no Brasil: mosaico de configurações e arena de controvérsias.

[CR8] Camurça, M (2019) Religião, política e espaço público no Brasil: perspectiva histórico/sociológica e a conjuntura das eleições presidenciais de 2018. Estudos de Sociologia, Recife, 2/2, pp 125–159

[CR9] Carranza B (2000). Renovação Católica Carismática: Origens, Mudanças E Tendências.

[CR10] Casanova J, Gabriel, Fischer, Yochi (2008). The problem of religion and the anxieties of European Secular Democracy in motzkin.

[CR11] Datta, N (2018) Modern-day crusaders in Europe. Tradition, family and property: analysis of a transnational, ultra-conservative, catholic-inspired influence network. Politicke Perspektive Doi 10.20901

[CR12] Dawson A, Rowland C (2007). The origins and character of the base ecclesial community: a Brazilian perspective. The Cambridge companion to liberation theology.

[CR13] Dias R (1996). Imagens de Ordem: A Doutrina Católica sobre a autoridade no Brasil (1922–1933).

[CR14] Ghiraldelli P (2020). A República Brasileira: de Deodoro a Bolsonaro.

[CR15] Gramsci A (1966) A Concepção Dialética da História. Civilização Brasileira, Rio de Janeiro

[CR16] Introvigne M (2016). Tradition, family and property (TFP) and the heralds of the gospel. The religious economy of Brazilian conservative Catholicism. Alternative Spirituality and Religion Review.

[CR17] Klaiber, J (1998) The church, dictatorships and democracy in Latin America. Eugene (Oregon), Wipf & Stock

[CR18] Levitsky S, Ziblatt D (2018). How democracies die.

[CR19] Machado, MDC (2012) Religião, Cultura e Política. Religião & Sociedade, Rio De Janeiro, 32/2, pp 29–56

[CR20] Mariano R, Gerardi D (2019). A América Latina em 2018 e ativismo político de evangélicos conservadores.

[CR21] Romano, R (1979) Brasil: Igreja Contra Estado (Crítica Ao Populismo Católico). São Paulo, Kayrós

[CR22] Rowland C (2007). The Cambridge companion to liberation theology.

[CR23] Silveira, ES (2016) O Evangelho dos Produtos Canção Nova: salvação, consumo e mídia eletrônica. Estudos Teológicos (Online), 56, pp 420–434, 2016

[CR24] Silveira ES da (2018) Glossolalias, justiça social e báculos episcopais - narrativas míticas entre carismáticos, progressistas e conservadores. In: da Silveira ES, Sampaio DS (eds) Narrativas Míticas: análise das histórias que as religiões contam. Petrópolis, Editora Vozes, pp 25–70

[CR25] Silveira ES (2019) da O evangelho dos produtos Canção: salvação, consumo e mídia eletrônica. Estudos Teologicos (Online), São Leopoldo, 56/2, pp 420-434 Available via http://periodicos.est.edu.br/index.php/estudos_teologicos/article/view/2422

[CR26] Sofiati FM (2012). Religião e Juventude: os Novos Carismáticos.

[CR27] Souza JJV (2002). Círculos Operários: A Igreja Católica e o mundo do trabalho no Brasil.

[CR28] Werneck, GL and Carvalho, MS (2020) A pandemia de COVID-19 no Brasil: crônica de uma crise sanitária anunciada Cadernos de Saúde Pública [online], vol.36, n. 5 10.1590/0102-311x00068820 32402007

[CR29] Zanotto G (2012). TFP. Tradição, Família e Propriedade. As idiossincrasias de um movimento católico no Brasil (1960-1995).

[CR30] Zanotto G, Caldeira RC (2014). Facetas do Tradicionalismo Católico.

